# Dietary Spermidine Mitigates Radiation‐Induced Intestinal Injury by Reshaping the Microbiota‐Barrier‐Inflammation Axis

**DOI:** 10.1002/fsn3.71924

**Published:** 2026-06-18

**Authors:** Song Li, Yanjiang Liu, Minglin Jiang, Yu Zha, Chang Liu, Hongtao Luo, Kejian Pan, Tao Zhang, Xiaolin Ren

**Affiliations:** ^1^ School of Laboratory Medicine Chengdu Medical College Chengdu China; ^2^ School of Basic Medical Sciences Chengdu Medical College Chengdu China; ^3^ School of Bioscience and Technology Chengdu Medical College Chengdu China; ^4^ The Second Affiliated Hospital of Chengdu Medical College (Nuclear Industry 416 Hospital) Chengdu China; ^5^ Irradiation Preservation and Effect Key Laboratory of Sichuan Province Chengdu China

**Keywords:** gut microbiota, Nrf2, radiation‐induced intestinal injury, spermidine

## Abstract

Radiation‐induced intestinal injury (RIII) is a major complication of radiotherapy, characterized by oxidative stress, intestinal barrier disruption, and gut microbiota dysbiosis. There is an urgent clinical need for highly effective and low‐toxicity radioprotective agents. Spermidine (SPD) is a natural polyamine widely found in various foods with well‐documented health‐promoting properties, yet its role in RIII remains elusive. Using a mouse model of whole‐abdominal irradiation, we demonstrated that SPD intervention significantly improved survival rates and mitigated intestinal pathological damage, including crypt loss and villus shortening. Mechanistically, SPD pretreatment activated the Nrf2 signaling pathway, thereby alleviating oxidative stress and reducing DNA double‐strand breaks in intestinal epithelial cells. Furthermore, SPD preserved intestinal barrier integrity by enhancing the expression of tight junction proteins (Occludin and ZO‐1) and reduced systemic inflammation (serum IL‐6). 16S rRNA sequencing revealed that SPD prevented radiation‐induced gut dysbiosis by significantly enriching beneficial butyrate‐producing bacteria (e.g.*, Lachnospiraceae_NK4A136_group*) while suppressing potential pathogens (e.g.*, Parabacteroides and Mucispirillum*). Our study reveals that SPD exerts multi‐targeted protection against RIII by coordinately regulating the “microbiota‐barrier‐inflammation” axis, positioning it as a promising candidate for preventing and treating RIII.

## Graphical abstract

Spermidine (SPD) protects against radiation‐induced intestinal injury by activating the Nrf2 pathway to reduce oxidative stress and DNA damage, enhancing tight junction proteins to restore barrier integrity, and remodeling gut microbiota (enriching butyrate‐producing bacteria while suppressing pathogens). These coordinated effects on the “microbiota‐barrier‐inflammation” axis improve survival and intestinal pathology.

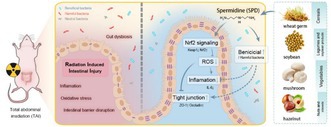

## 1 Introduction

Currently, radiation therapy constitutes a core component of comprehensive treatment regimens for malignant tumors in the abdominal and pelvic regions (e.g., colorectal cancer, cervical cancer), with over half of patients undergoing such therapy during treatment (De Ruysscher et al. 2019). However, while destroying tumor tissue, ionizing radiation inevitably damages adjacent normal intestinal tissue. The resulting radiation‐induced intestinal injury (RIII) has become a major clinical challenge affecting treatment efficacy and patient quality of life (Zhao et al. 2025). Characteristic pathological features of RIII include disruption of the intestinal epithelial barrier, oxidative stress due to excessive reactive oxygen species (ROS) production, and gut microbiota dysbiosis. Clinically, patients present with persistent diarrhea, abdominal pain, and even bloody stools. In severe cases, the condition can progress to intestinal perforation and sepsis, posing a life‐threatening risk (Chaves‐Pérez et al. 2019; Xie et al. 2024; Zhang, Tang, et al. 2024; Zhang, Xu, et al. 2024; Zhang, Zhao, et al. 2024).

Current therapeutic options for RIII remain limited. While traditional radioprotective agents like amifostine have been clinically used as cytoprotectants to mitigate DNA damage from chemotherapy or radiotherapy (Kouvaris et al. 2007), their toxicity and side effects restrict widespread application (Ji et al. 2023; King et al. 2020; Singh and Seed 2019). Consequently, developing highly effective and specific strategies for protecting against radiation‐induced intestinal injury remains a critical unmet clinical need.

In recent years, the gut microbiota has emerged as a complex “functional organ”, playing increasingly prominent roles in maintaining intestinal health and regulating local and systemic inflammatory responses (Jian et al. 2021; Wang, Wang, et al. 2025; Wang, Xu, et al. 2025; Xin et al. 2022). Growing evidence indicates that radiation exposure causes severe disruption of gut microbiota structure, characterized by depletion of beneficial commensal bacteria and overgrowth of potential pathogens. This dysbiosis further compromises intestinal barrier function, amplifying persistent inflammatory cascades and exacerbating the pathological progression of RIII (Li et al. 2021). Therefore, intervening in the gut microbiota to break the vicious cycle of “dysbiosis‐barrier damage‐inflammation amplification” offers a highly promising new approach for the prevention and treatment of RIII.

Spermidine (SPD) is a naturally occurring polyamine widely present in foods such as wheat germ, exhibiting multiple biological activities including antioxidant, anti‐inflammatory, and anti‐apoptotic biological activities (Madeo et al. 2018; Niechcial et al. 2023). Previous studies have demonstrated that SPD effectively alleviates intestinal inflammation in models of inflammatory bowel disease (IBD) and other intestinal inflammatory diseases. Its protective effects involve enhancing barrier function, suppressing oxidative stress, and modulating the immune microenvironment (Jiang et al. 2023; Ma et al. 2020). SPD's antioxidant effects are closely associated with the activation of the nuclear factor E2‐related factor 2 (Nrf2) signaling pathway. SPD promotes Nrf2 nuclear translocation by inhibiting its negative regulator Keap1, thereby initiating the expression of a series of endogenous antioxidant enzymes—a core mechanism for cellular defense against oxidative damage (Gobert et al. 2018; Wen et al. 2019; Yan et al. 2023). Notably, RIII and IBD exhibit high similarity in key pathological features such as intestinal barrier disruption, dysbiosis, and chronic inflammation (Askari et al. 2023; Lu et al. 2023). We hypothesize that SPD may exert similar protective effects in RIII by modulating this shared pathological axis.

Against this backdrop, this study systematically evaluated the protective effects of SPD against radiation‐induced intestinal injury and investigated whether its actions correlate with three mechanisms: repairing the intestinal barrier, suppressing oxidative stress and DNA damage via the Keap1/Nrf2 pathway, and restoring microbiota homeostasis. The findings provide experimental evidence for SPD's development as a protective agent against radiation‐induced intestinal injury and further elucidate the role of the “microbiota‐barrier‐inflammation” axis in RIII pathogenesis.

## 2 Materials and Methods

### 2.1 Reagents

Spermidine trihydrochloride (CAS: 334‐50‐9) was purchased from Shanghai Macklin Biochemical Co. Ltd. For in vitro experiments, Spermidine was dissolved in serum‐free RPMI 1640 medium (Wuhan Procell Life Technology Co. Ltd.) to prepare a 10 mM stock solution. The solution was aliquoted and stored at −20°C. For in vivo experiments, Spermidine was dissolved in sterile physiological saline immediately prior to use.

### 2.2 Mice and Feeding Conditions

All animal experiments were approved by the Animal Ethics Committee of Chengdu Medical College (Approval No. [2025] 64; approval date: July 27, 2025). This approval specifically covered the experimental procedures described in this study, including total abdominal irradiation, SPD administration, and subsequent tissue, blood, and fecal sample collection. Animal care and experimental protocols were conducted in accordance with the national regulations and institutional guidelines for animal care and use in China. Male C57BL/6J mice (6–8 weeks old) were obtained from Chengdu Dashuo Laboratory Animal Co. Ltd. The mice were maintained under specific pathogen‐free (SPF) conditions at a temperature of 22°C–24°C with a 12 h/12 h light–dark cycle and had free access to food and water.

### 2.3 Cell Culture

The rat intestinal crypt epithelial cell line IEC‐6 was acquired from the Cell Resource Center of the Shanghai Institute of Life Sciences, Chinese Academy of Sciences. The cells were maintained at 37°C in a 5% CO_2_ incubator using RPMI 1640 medium supplemented with 10% (v/v) heat‐inactivated fetal bovine serum (Gibco, USA) and 1% (v/v) penicillin–streptomycin (Beyotime Biotechnology, China).

In vitro irradiation was performed using an X‐ray irradiator (Rad Source Technologies, USA). IEC‐6 cells received a single 10 Gy dose at a dose rate of 150 cGy/min (voltage 225 kV, current 12.5 mA, source‐skin distance 50 cm, 2.0 mm Al filter) following corresponding pretreatments. This dose was selected for establishing the radiation‐induced intestinal epithelial cell injury model in IEC‐6 cells, which could stably induce significant oxidative stress, DNA damage, and barrier dysfunction without excessive cell death and has been widely used in the field of radiation enteritis research (Wang, Wang, et al. 2025; Wang, Xu, et al. 2025; Zhang et al. 2020; Zhou et al. 2022).

### 2.4 Animal Irradiation, Survival, and Sample Collection

In vivo irradiation was performed using the same X‐ray irradiator and parameters as described in Section 2.3, except for the radiation dose. All irradiations were delivered as a single total abdominal exposure (single fraction). Two independent irradiation protocols were used for different experimental endpoints.

Protocol A (14 Gy TAI) was designed for survival analysis. Mice were randomly divided into four groups: Control (*n* = 6), IR (*n* = 10), High‐dose SPD (HSPD, 30 mg/kg, *n* = 10), and Low‐dose SPD (LSPD, 5 mg/kg, *n* = 10). SPD or saline was administered once daily by oral gavage for 7 consecutive days before irradiation. The total amount administered was 210 mg/kg for the HSPD group (30 mg/kg × 7 days) and 35 mg/kg for the LSPD group (5 mg/kg × 7 days). Survival was monitored daily for 15 days post‐irradiation.

Protocol B (15 Gy TAI) was designed for histopathological and molecular analysis. A separate cohort of mice received 15 Gy TAI to induce more pronounced intestinal damage. SPD (30 mg/kg) or saline was administered once daily by oral gavage for 7 consecutive days before irradiation. The sample sizes varied by assay: colon length measurement (*n* = 5 per group); histological and immunohistochemical analyses (*n* = 3 per group); serum cytokine detection (*n* = 3 per group); 16S rRNA sequencing (*n* = 5 per group). Mice were euthanized on day 3 post‐irradiation, and colon tissue, serum, and fecal samples were collected for subsequent analysis.

### 2.5 Enzyme‐Linked Immunosorbent Assay (ELISA)

Blood samples were collected via eyeball puncture. Serum was separated by centrifugation at 4000 rpm for 20 min after overnight incubation at 4°C. The concentration of interleukin‐6 (IL‐6) was measured using a commercial mouse IL‐6 ELISA kit (Ruixin Biotechnology Co. Ltd.) according to the manufacturer's instructions and established protocols (Cai et al. 2022). Absorbance was measured at 450 nm using a microplate reader (Molecular Devices, USA), and IL‐6 concentrations were calculated based on the standard curve.

### 2.6 Immunohistochemistry

Paraffin‐embedded intestinal tissue sections underwent dewaxing, rehydration, antigen retrieval, and endogenous peroxidase blocking. Primary antibodies against ZO‐1 or Occludin were incubated overnight at 4°C, followed by staining with an HRP‐labeled secondary antibody/DAB chromogenic system according to standard immunohistochemistry protocols (Cho et al. 2026). Sections were counterstained with hematoxylin and mounted for observation.

### 2.7 Cell Viability and Cytotoxicity Assay (CCK‐8)

The potential toxicity of SPD on IEC‐6 cells was assessed using the CCK‐8 assay. Cells in the logarithmic growth phase were seeded in a 96‐well plate at a density of 1 × 10^4^ cells per well. After attachment, cells were treated with fresh medium containing SPD at concentrations ranging from 0 to 64 μM for 24 h. Subsequently, 10 μL of CCK‐8 solution (Biyuntian, China) was added to each well, followed by incubation at 37°C for 2 h. The absorbance of each well was measured at 450 nm using a microplate reader (Biosharp, China). Based on the results, 4 μM was identified as the non‐cytotoxic working concentration for subsequent experiments.

### 2.8 Immunofluorescence

For immunofluorescence, IEC‐6 cells were seeded on coverslips and pretreated with 4 μM SPD in complete medium for 24 h. After pretreatment, cells were exposed to a single 10 Gy Xray irradiation (see Section 2.3). Following irradiation, cells were further incubated for 6 or 12 h, then fixed with 4% paraformaldehyde, permeabilized with 0.1% Triton X‐100, and blocked with BSA, following standard immunofluorescence staining protocols (Deng et al. 2025). After overnight incubation at 4°C with a γ‐H2AX primary antibody, samples were incubated with a fluorescent secondary antibody and counterstained with DAPI, then visualized under a fluorescence microscope.

### 2.9 Western Blot Analysis

IEC‐6 cells were seeded into 6 cm dishes at 1 × 10^6^ cells per dish and pretreated with 4 μM SPD in complete medium for 24 h. Cells were then exposed to a single 10 Gy X‐ray irradiation (see Section 2.3). Following irradiation, cells were further incubated for 6 h. Total proteins were extracted using RIPA lysis buffer supplemented with protease and phosphatase inhibitors. Protein concentrations were determined using a BCA protein assay kit (Thermo Fisher Scientific, USA). Equal amounts of protein were separated by 10% SDS‐PAGE and electrotransferred onto 0.22 μm PVDF membranes (Merck Millipore, Germany). The membranes were blocked with 5% non‐fat milk at room temperature for 1 h and subsequently incubated with primary antibodies against γ‐H2AX (Abcam, 1:1000) and HRP‐conjugated secondary antibodies (Proteintech, 1:5000). Finally, the blots were developed using an ECL chemiluminescent substrate (Biosharp, China) and images were captured using a gel imaging system (Bio‐Rad, USA). β‐Actin was used as an internal control for quantitative analysis of band intensities. All procedures were performed in accordance with established Western blot protocols (Ha et al. 2025).

### 2.10 Intracellular Reactive Oxygen Species (ROS) Detection

IEC‐6 cells were pretreated with 4 μM SPD for 24 h as described in Section 2.9, then exposed to a single 10 Gy X‐ray irradiation (see Section 2.3). Following irradiation, cells were further incubated for 1 h. Intracellular ROS levels were assessed using a commercial ROS detection kit (Biosharp, China). Briefly, cells were incubated with 10 μM DCFH‐DA probe at 37°C in the dark for 30 min, then collected, and ROS levels were quantified by flow cytometry as previously described (Eruslanov and Kusmartsev 2010).

### 2.11 Microbiome Analysis

Mouse fecal samples were collected and stored at −80°C. Genomic DNA extraction, 16S rRNA gene sequencing, and primary bioinformatics analysis were performed by Shanghai OE Biotech Co. Ltd. The QIIME2 (2020.11) workflow was used for data quality control, denoising, and generation of an Amplicon Sequence Variant (ASV) table. Subsequent statistical analysis of α‐diversity (ACE, Chao1, Shannon, Simpson indices), β‐diversity (PCoA based on Bray‐Curtis distance), and intergroup species differences was conducted using R software (v4.0.3). Microbiome data processing was performed following the QIIME2 pipeline as described (Estaki et al. 2020).

### 2.12 Statistical Analysis

Results are presented as the mean ± standard error of the mean (SEM) from at least three independent experiments. Data were analyzed using GraphPad Prism version 10.4 (GraphPad Software, USA). Comparisons between two groups were performed using unpaired *t*‐tests, while comparisons among three or more groups were conducted using one‐way analysis of variance (ANOVA) followed by Tukey's multiple comparison test. Statistical significance was set at *p < 0.05, **p < 0.01, ***p < 0.001.

## 3 Results

### 3.1 Effect of SPD on Survival, Intestinal Pathology, and Systemic Inflammation in Irradiated Mice

**FIGURE 1:**
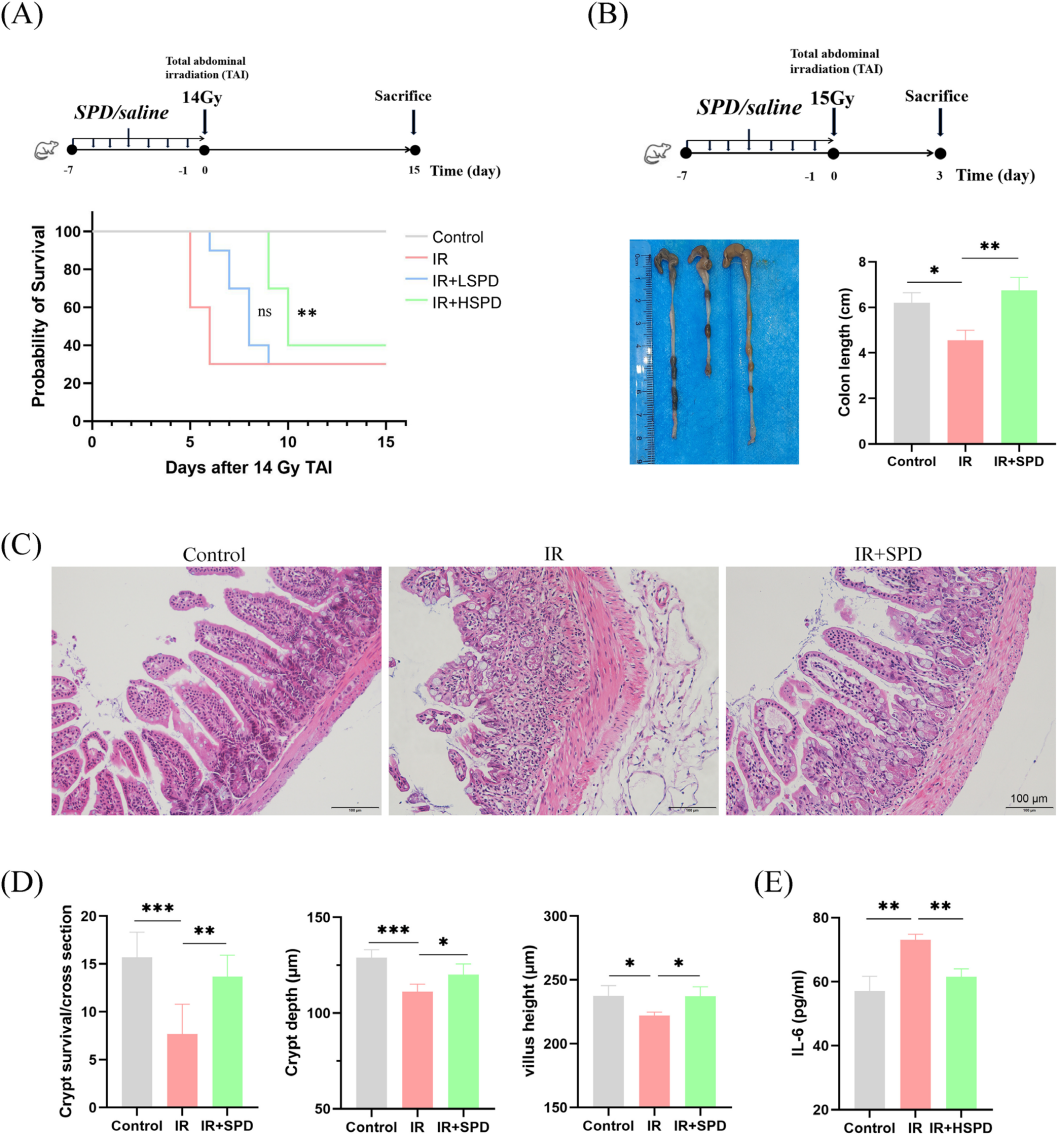
SPD ameliorates radiation‐induced intestinal injury and systemic inflammation in mice. (A) Survival curves of mice following 14 Gy TAI with or without SPD pretreatment (*n* = 6 for the control group and *n* = 10 for the model and treatment groups). Significance comparisons shown are versus the IR group. (B) Representative images and quantification of colon length after 15 Gy TAI (*n* = 3). (C) Representative H&E‐stained sections of the small intestine from mice after 15 Gy TAI, showing the damage to crypts and villi and the protective effect of SPD (*n* = 6). (D) Quantitative analysis of the number of surviving crypts, crypt depth, and villus length, confirming the improvement in intestinal histoarchitecture with SPD treatment. (E) Serum levels of the pro‐inflammatory cytokine IL‐6, demonstrating that SPD suppresses radiation‐induced systemic inflammation. *p < 0.05, **p < 0.01, ***p < 0.001.

To evaluate the systemic protective effect of SPD against radiation‐induced intestinal injury, we established a mouse total abdominal irradiation (TAI) model. Survival analysis revealed that in the 14 Gy irradiation model, high‐dose SPD (30 mg/kg, HSPD) significantly improved the survival rate (*p* < 0.01) compared with the IR model group, while low‐dose SPD (5 mg/kg, LSPD) did not significantly reduce mortality but markedly prolonged the median survival time (Figure 1A). Given the superior protective effect of the HSPD group, this dose was used in subsequent in vivo experiments. In the 15 Gy irradiation model, IR mice exhibited typical colon shortening, which was effectively attenuated by SPD pretreatment (*p* < 0.01, Figure 1B). Further histological evaluation confirmed that IR caused severe disruption of crypt architecture and extensive villus loss. In contrast, SPD‐pretreated small intestinal tissue maintained better structural integrity, with preserved crypts and villi (Figure 1C). Quantitative analysis of villus length, crypt depth, and the number of surviving crypts demonstrated significant improvement in these parameters following SPD intervention (Figure 1D).

Since intestinal barrier damage often leads to systemic inflammation, we next determined whether the pathological improvement conferred by SPD translated into a reduction in systemic inflammatory markers. The data showed that irradiation significantly elevated serum IL‐6 levels, while SPD treatment markedly suppressed this increase (*p* < 0.01, Figure 1E).

### 3.2 Effect of SPD on Intestinal Physical Barrier and Tight Junction Protein Expression

**FIGURE 2:**
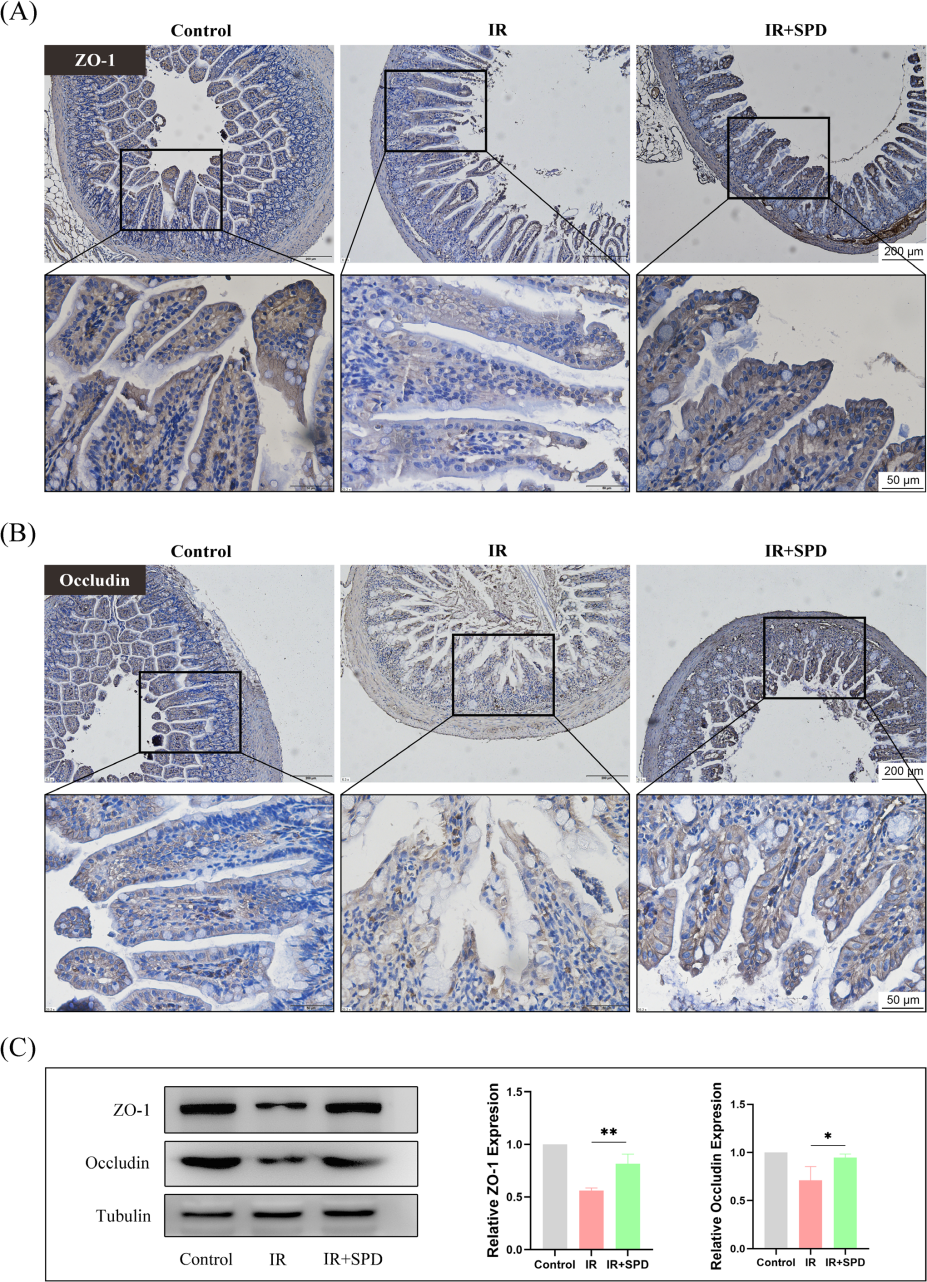
SPD upregulates tight junction proteins in vivo and in vitro. Representative immunohistochemical images of the core tight junction proteins (A) ZO‐1 and (B) Occludin in mouse intestinal tissues after irradiation with or without SPD treatment. (C) Western blot analysis of ZO‐1 and Occludin expression in IEC‐6 cells following irradiation and SPD pretreatment. *p < 0.05, **p < 0.01.

Having observed that SPD ameliorates structural damage to the intestinal epithelium, we sought to determine whether this protective effect extends to the molecular level of barrier function. Since the integrity of the intestinal barrier is critically maintained by tight junction complexes, we analyzed the expression and localization of its core proteins, ZO‐1 and Occludin. Immunohistochemistry revealed that irradiation severely disrupted the expression and membrane localization of ZO‐1 and Occludin compared with the control group (Figure 2A,B). SPD treatment largely preserved the continuous, membrane‐associated expression patterns and protein levels of these tight junction proteins. This protective effect was corroborated in vitro, where SPD similarly upregulated the expression of ZO‐1 and Occludin in IEC‐6 cells (Figure 2C).

### 3.3 Effect of SPD on Radiation‐Induced DNA Damage, Oxidative Stress, and the Keap1/Nrf2 Pathway in IEC‐6 Cells

**FIGURE 3:**
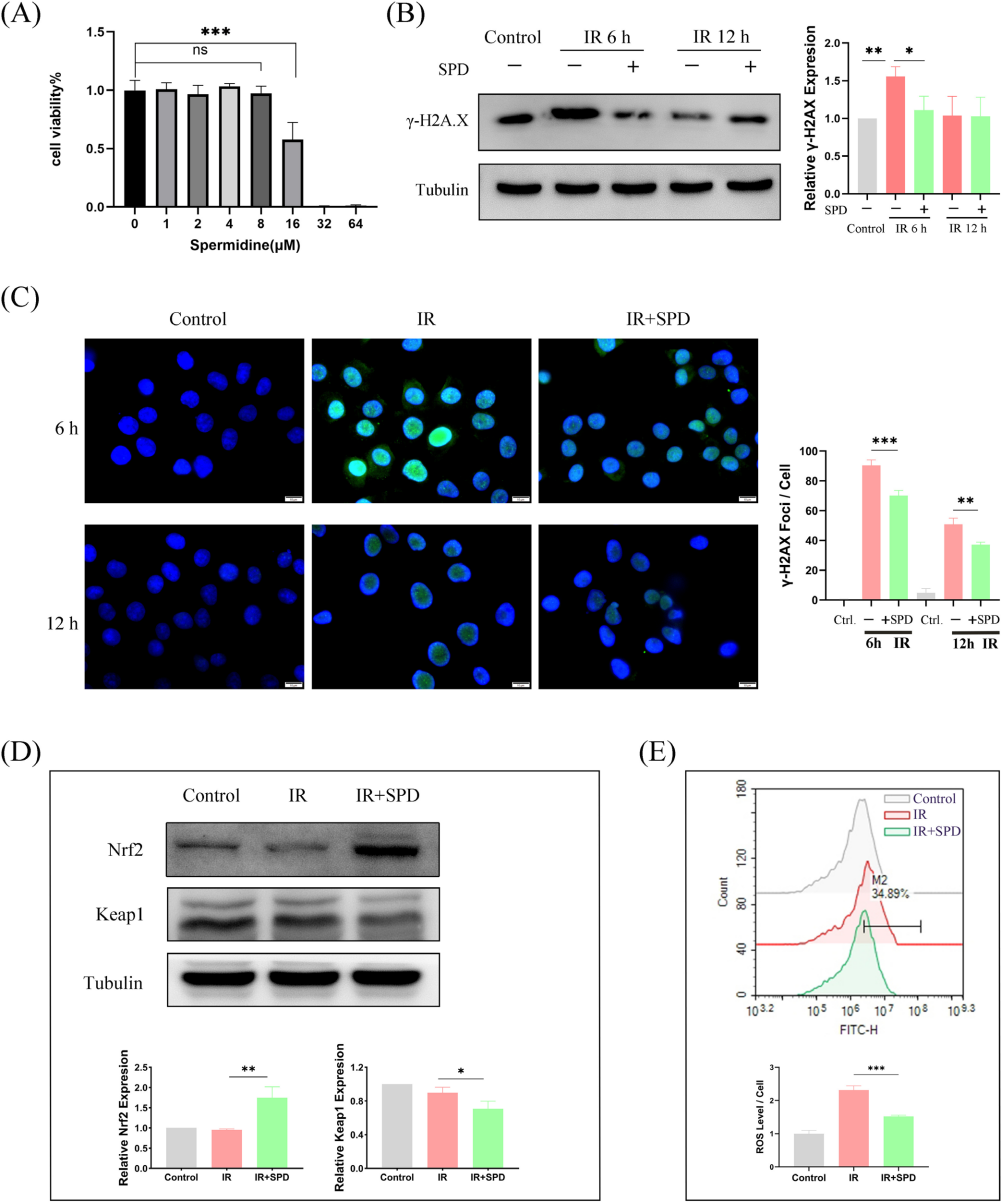
SPD alleviates oxidative stress and DNA damage by activating the Keap1/Nrf2 pathway. (A) Determination of a non‐cytotoxic working concentration (4 μM) of SPD in IEC‐6 cells using the CCK‐8 assay. (B) Reduction of radiation‐induced γ‐H2AX protein expression by SPD pretreatment, as quantified by western blot analysis. (C) Attenuation of radiation‐induced γ‐H2AX foci formation by SPD, observed by immunofluorescence staining (γ‐H2AX foci in green, nuclei counterstained with DAPI in blue). (D) Activation of the Keap1/Nrf2 pathway by SPD, demonstrated by increased Nrf2 and decreased Keap1 protein levels (western blot). (E) Attenuation of radiation‐induced intracellular ROS accumulation by SPD, measured by flow cytometry. *p < 0.05, **p < 0.01, ***p < 0.001.

Beyond protecting barrier function, SPD exerted multifaceted protective effects. To delineate its role in combating oxidative stress, a primary insult in RIII, the study first determined a safe working concentration. In IEC‐6 cells, the CCK‐8 assay identified 4 μM SPD as non‐cytotoxic, in contrast to the inhibitory effect at 16 μM SPD (*p* < 0.001, Figure 3A), and 4 μM SPD was used for all subsequent experiments.

The effect of SPD on radiation‐induced DNA damage was then examined. SPD pretreatment effectively reduced the levels of γ‐H2AX, a marker of DNA double‐strand breaks, as shown by both western blot (Figure 3B) and a decrease in fluorescent foci (Figure 3C). Mechanistic investigation revealed that SPD treatment activated the Keap1/Nrf2 antioxidant pathway, promoting Nrf2 expression while suppressing Keap1 (Figure 3D). Consistent with this, SPD pretreatment significantly attenuated radiation‐induced ROS bursts (*p* < 0.001, Figure 3E).

### 3.4 Effect of SPD on Radiation‐Induced Gut Dysbiosis and Microbial Diversity

To further delineate the broader protective profile of SPD, we investigated its impact on the gut microbiota, a key regulator of intestinal homeostasis that interacts closely with host barrier and immune function. We hypothesized that the protection of the host intestinal environment by SPD might be paralleled by a beneficial reshaping of the microbial community. To comprehensively assess this, we performed 16S rRNA gene sequencing.

**FIGURE 4:**
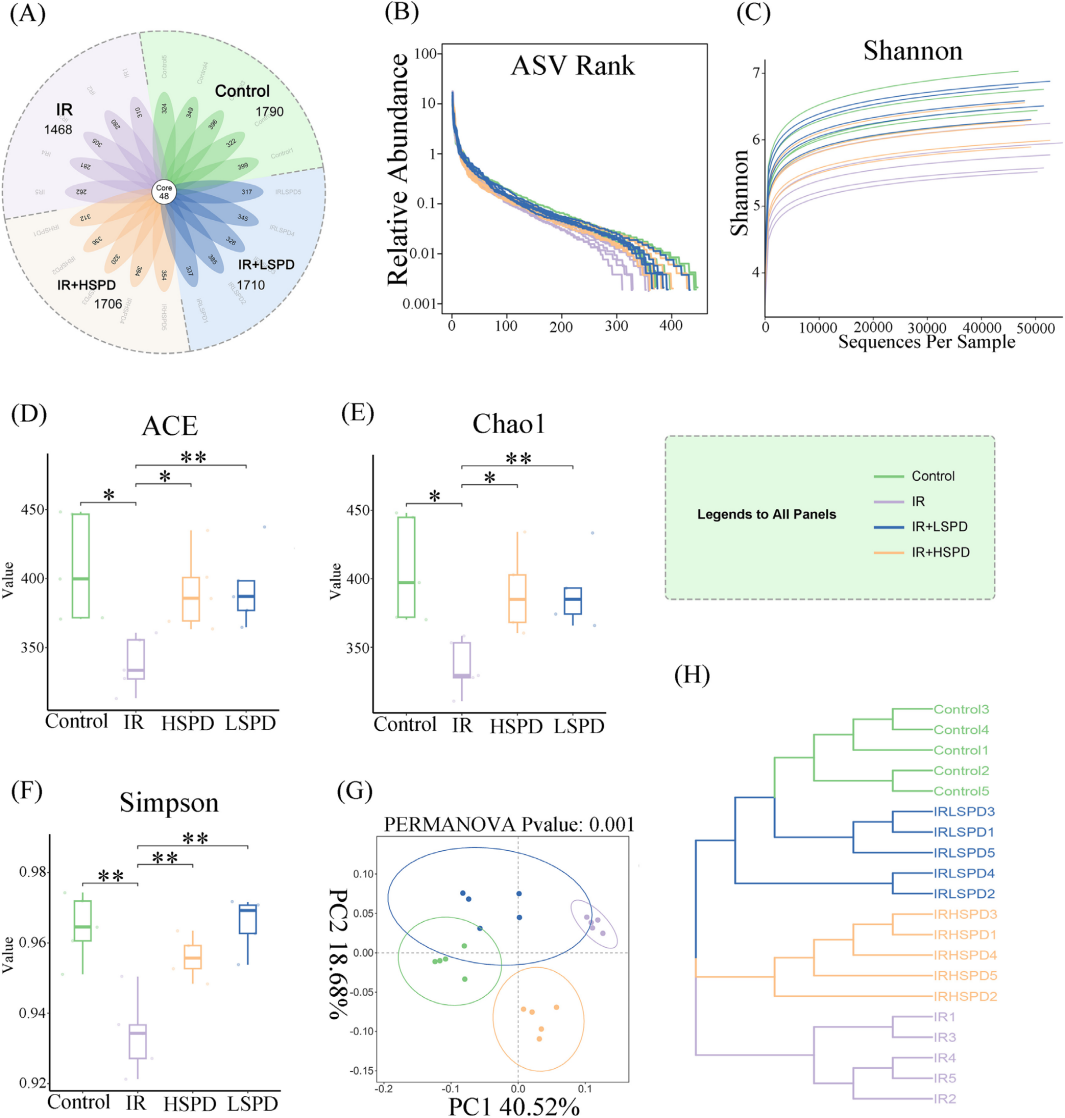
SPD prevents radiation‐induced gut microbial dysbiosis (A) Flower plot showing ASV overlap among groups. (B) Rank‐abundance curve for sequencing coverage validation. (C) Shannon rarefaction curve confirming sufficient sequencing depth. (D–F) ACE, Chao1, and Simpson indices of gut microbial α‐diversity. (G) PCoA of microbial community structure. (H) UPGMA hierarchical clustering tree of sample similarity. (*n* = 5 per group) *p < 0.05, **p < 0.01.

Initial analysis of operational taxonomic units (OTUs) revealed that radiation significantly reduced microbial species abundance, a loss that was prevented by SPD treatment (Figure 4A). The Rank Abundance curves (Figure 4B) and Shannon curves (Figure 4C) showed flattening trends, confirming that the majority of microbial diversity had been captured.

We next assessed community richness and evenness using α‐diversity indices. Compared with the control group, the IR group exhibited significant reductions in the ACE index (Figure 4D) and Chao1 index (Figure 4E), as well as a decrease in the Simpson index (Figure 4F), indicating that radiation disrupted both microbial richness and evenness. SPD intervention (LSPD and HSPD) significantly restored these α‐diversity metrics toward control levels. β‐Diversity analysis based on Bray‐Curtis distance showed that samples from the control, IR, and SPD‐treated groups formed distinct clusters in principal coordinates analysis (PCoA; Figure 4G). The IR group samples clearly separated from the control group, whereas the SPD‐treated groups shifted toward the control cluster. PERMANOVA testing confirmed significant differences in community structure among groups. Consistently, UPGMA hierarchical clustering tree (Figure 4H) verified distinct grouping, with SPD‐treated groups clustering near the control.

### 3.5 Effect of SPD on Gut Microbiota Composition

**FIGURE 5:**
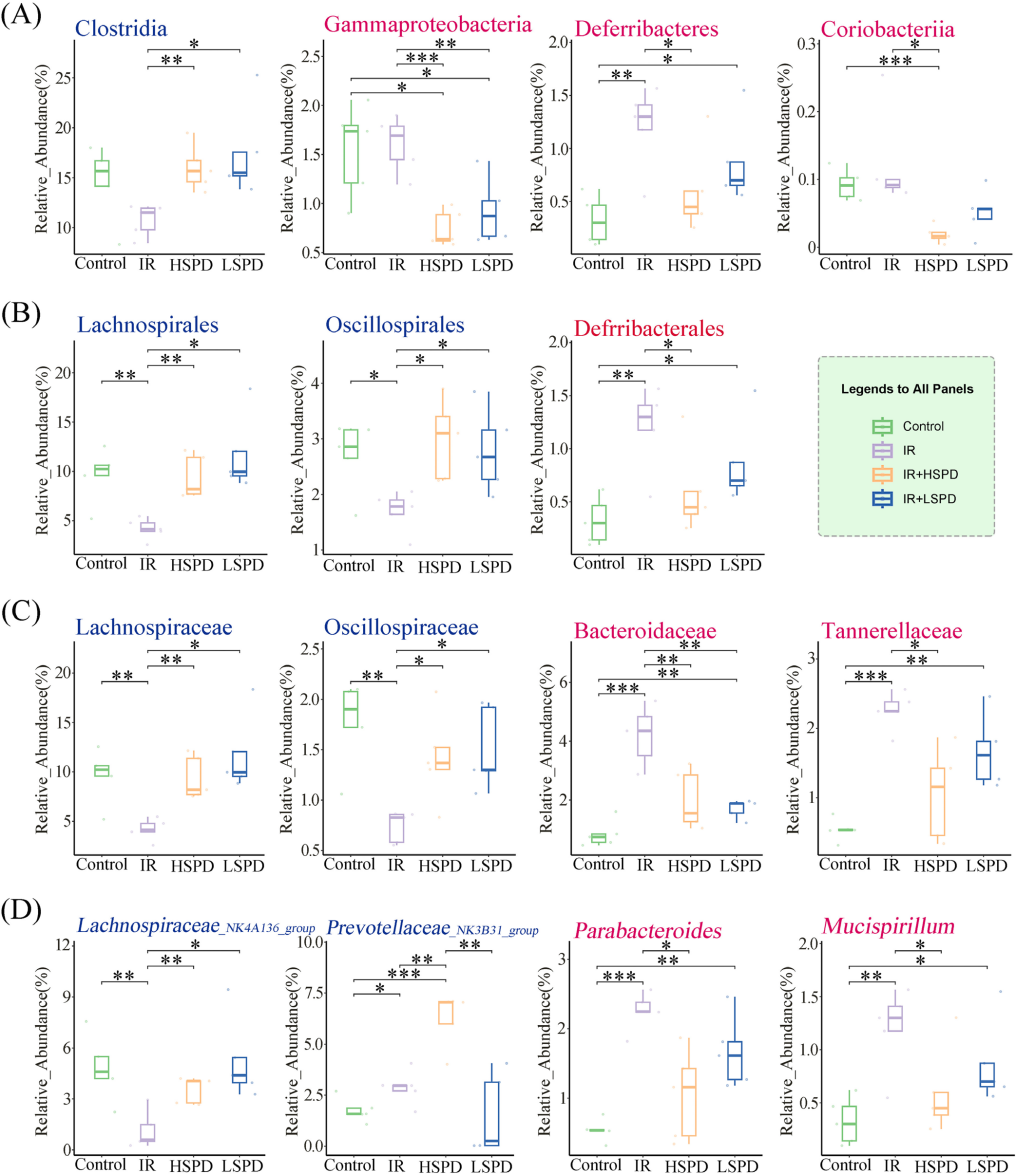
SPD specifically modulates gut microbiota composition by enriching beneficial and suppressing pathogenic bacteria. Differential analysis of representative bacterial taxa at the (A) class, (B) order, (C) family, and (D) genus levels demonstrates the consistent modulatory effects of SPD. Beneficial bacterial names are shown in blue; pathogenic bacterial names are shown in red. *p < 0.05, **p < 0.01, ***p < 0.001.

The protective effect of SPD on microbial diversity and structure prompted an investigation into the specific bacterial taxa responsible for these changes. Analysis across multiple taxonomic levels revealed consistent modulatory effects of SPD, with the differential analysis of representative taxa across these levels presented in Figure 5. For example, SPD prevented a radiation‐induced decline in the class Clostridia, which contains many beneficial symbionts, as well as an increase in the class Deferribacteres, a group known to harbor pro‐inflammatory genera. Beyond these broad taxonomic shifts, SPD's modulatory effects were consistently significant down to the order, family, and genus levels. Of note, the modulatory effect of SPD was dose‐dependent for many bacterial taxa, and this dose‐dependency was particularly evident in the suppression of pro‐inflammatory and pathogenic taxa (e.g., Deferribacteres, *Parabacteroides*, and *Mucispirillum*). Stacked bar charts depicting the microbial community composition at the aforementioned taxonomic levels are provided in Figure S1. We next focused our analysis on the genus level, where the most specific and functionally interpretable changes were identified.

A heatmap analysis of the 30 most significantly altered OTUs revealed a clear regulatory pattern (Figure S2): SPD selectively enriched beneficial commensals while suppressing pathogenic bacteria. For instance, SPD significantly increased the relative abundance of *Lachnospiraceae_NK4A136_group*, a core butyrate producer whose metabolites are crucial for intestinal epithelial energy homeostasis, tight junction integrity, and anti‐inflammation. Conversely, SPD downregulated the abundance of multiple inflammation‐associated and potentially pathogenic genera, including *Parabacteroides* and *Mucispirillum*. These pathogens are known to disrupt the epithelial barrier and activate pro‐inflammatory signaling through the release of endotoxins such as lipopolysaccharide (LPS).

In summary, SPD does not exert a broad, indiscriminate effect on the gut microbiota. Instead, it precisely prevents radiation‐induced dysbiosis through a dual mechanism of beneficial bacteria enrichment combined with pathogenic bacteria suppression, thereby steering the microbial ecosystem toward a state that supports host protection and maintenance of homeostasis.

## 4 Discussion

Radiation‐induced intestinal injury (RIII) is a major complication of abdominal and pelvic tumor radiotherapy, affecting over 50% of patients with varying degrees of intestinal damage. Typical clinical manifestations include persistent diarrhea and bloody stools, significantly impairing patients' quality of life and treatment progress (Ahmad et al. 2012; Barnett et al. 2009; Delaney et al. 2005). Current clinical interventions for RIII are limited, necessitating the urgent development of novel protective strategies that balance efficacy and safety. SPD, a natural polyamine, has demonstrated protective effects in inflammatory bowel disease through ROS scavenging, upregulation of tight junction proteins, and enrichment of beneficial bacteria (Guo et al. 2005; Qiu et al. 2022; Zhang, Tang, et al. 2024; Zhang, Xu, et al. 2024; Zhang, Zhao, et al. 2024). In the present study, we demonstrated that SPD significantly improved the survival of irradiated mice and alleviated intestinal damage, including colon shortening, crypt loss, and villus atrophy. SPD also reduced serum IL‐6 levels, indicating mitigation of systemic inflammation. Consistent across tissue and cellular levels, SPD preserved the intestinal physical barrier by upregulating tight junction proteins (ZO‐1 and Occludin). Furthermore, SPD remodeled the global gut microbiota composition, promoting its maintenance toward a state similar to that of unirradiated controls.

We first investigated the cellular mechanisms underlying SPD's protective effects. Our results demonstrate that SPD protects intestinal epithelial cells by activating the Keap1/Nrf2 pathway to scavenge ROS and reduce DNA damage. Further mechanistic investigation revealed that SPD's potent antioxidant effects are closely linked to its precise regulation of the Keap1/Nrf2 axis (Wen et al. 2019). Radiation‐induced oxidative stress leads to substantial intracellular ROS accumulation, and SPD intervention may modify the sensor cysteine residues on Keap1 protein via its inherent electrophilic properties, thereby disrupting Keap1‐mediated constitutive degradation of Nrf2. Nrf2 stabilization and nuclear translocation subsequently initiate transcriptional programs for a battery of endogenous antioxidant enzymes and phase II detoxification enzymes, including HO‐1 and NQO1 (Imazu et al. 2024). This activation of a systemic cellular defense constitutes the core molecular basis for SPD's efficacy in scavenging ROS, mitigating DNA damage (manifested as reduced γ‐H2AX foci), and ultimately protecting crypt stem cells.

Notably, Nrf2 activation extends beyond radical scavenging. Recent studies confirm that Nrf2 directly or indirectly upregulates the expression and assembly of intestinal tight junction proteins (e.g., Occludin; Ganapathy et al. 2023; Yu et al. 2024). Therefore, the observed promotion of ZO‐1 and Occludin expression by SPD in this study may stem not only from alleviated oxidative stress but also from direct transcriptional effects following sustained activation of the Nrf2 pathway. This reveals a synergistic, mutually beneficial mechanism through which SPD establishes a link between antioxidant defense and barrier repair via the Keap1/Nrf2 axis.

Beyond early antioxidant protection, SPD also exerts long‐term protective effects via the gut microbiota. Research indicates that the gut microbiota modulates local inflammation through the “microbiota‐immune axis” (Yi et al. 2023), and its dysregulation (e.g., pathogenic translocation) can directly amplify RIII pathological damage (Liu et al. 2021; Zhao et al. 2021). Notably, short‐chain fatty acids (SCFAs)—metabolic products of the microbiota—maintain intestinal barrier integrity by activating the GPR43 receptor (Dalile et al. 2019), providing a theoretical basis for preventing RIII through microbiota regulation. In our study, we found that irradiation significantly reduced the α‐diversity index of gut microbiota, with marked decreases in ACE index, Chao1 index, and other metrics. PCoA analysis indicated that the microbial structure in the IR group significantly diverged from the control group, while SPD intervention promoted its convergence toward the control pattern. Further analysis revealed that SPD continuously remodels gut microbiota structure, specifically by effectively suppressing multiple pro‐inflammatory and pathogenic bacteria including *Parabacteroides* and *Mucispirillum*, and significantly increasing the abundance of butyrate‐producing genera such as *Lachnospiraceae_NK4A136_group*, suggesting a potential promotion of short‐chain fatty acid (SCFA) production. Butyrate, serving as the primary energy source for intestinal epithelial cells, continuously enhances the expression of tight junction proteins ZO‐1 and Occludin by activating the AMPK signaling pathway, thereby reinforcing barrier integrity. Concurrently, this study observed SPD‐induced enrichment of SCFA‐producing bacteria in RIII, suggesting potential regulation of immune homeostasis through SCFAs/GPR signaling pathways (Ge et al. 2024; Ma et al. 2020; Wu et al. 2023). Furthermore, long‐term suppression of pathogenic bacteria directly reduces the release of toxic substances such as lipopolysaccharide (LPS), helping to block the TLR4/NF‐κB inflammatory cascade and thereby mitigate the development of chronic enteritis following radiation exposure (Berry et al. 2015; Herp et al. 2021; Rastall 2004; Santoso et al. 2022).

SPD‐induced gut microbiota remodeling ultimately contributes to establishing a self‐sustaining metabolic‐immune homeostasis. Our data support a model in which SPD‐induced microbiota remodeling forms a virtuous cycle potentially initiated by increased short‐chain fatty acids (SCFAs): these metabolites promote intestinal barrier repair, improved barrier function leads to reduced inflammation, and the ameliorated inflammatory environment ultimately facilitates colonization resistance by beneficial bacteria, thereby further consolidating the microbial structure (Guo et al. 2020; Leibovitzh et al. 2022; Wang et al. 2024). Nevertheless, it should be noted that while our 16S rRNA sequencing data identified specific bacterial changes and inferred their functional association with SCFA production, this study did not directly measure SCFA levels. Furthermore, definitive causal evidence through approaches such as fecal microbiota transplantation (FMT) remains to be established in future studies.

The self‐sustaining positive feedback loop described above, which was maintained throughout the 15‐day observation period post‐irradiation, provides a key mechanistic basis for the improved survival observed in the high‐dose SPD group. At the molecular level, this virtuous cycle relies on SPD's precise regulation of microbiota‐immune interactions. Post‐irradiation dysbiosis abnormally activates the TLR4/NF‐κB pathway, significantly elevating serum levels of the pro‐inflammatory cytokine IL‐6. Consistent with this, we found that SPD intervention effectively reduces IL‐6 levels (Figure 1E). This effect is closely associated with SPD's ability to downregulate TLR4 ligands (e.g., LPS) through microbiota remodeling. Specifically, increased abundance of beneficial bacteria like *Prevotellaceae_NK3B31_group* synergized with other commensals to effectively suppress overgrowth of harmful bacteria such as *Parabacteroides* and *Mucispirillum*, thereby maintaining a balanced gut microbial ecology (Wang et al. 2024; Zhang, Tang, et al. 2024; Zhang, Xu, et al. 2024; Zhang, Zhao, et al. 2024). This complete metabolic‐immune positive feedback loop, when integrated with the early antioxidant defense conferred by the Nrf2 pathway, forms a temporally coordinated and functionally complementary protective system. This system not only explains SPD's efficacy during the acute phase but also elucidates its role in promoting long‐term homeostasis.

The therapeutic promise of SPD is further supported by its translational feasibility as a dietary intervention. In designing the present study, we selected two doses—5 and 30 mg/kg per day (administered once daily for 7 consecutive days)—based on published studies in which effective doses generally ranged from 2 to 50 mg/kg across different experimental models (Liao et al. 2021; Ma et al. 2020; Morselli et al. 2011; Singh et al. 2021). The total dose administered over the 7‐day period was 35 mg/kg for the low‐dose group and 210 mg/kg for the high‐dose group. The low dose was chosen because its human equivalent can be achieved through daily intake of SPD‐rich foods (e.g., wheat germ, soybeans, mushrooms), whereas the high dose would require dietary supplementation on top of regular food intake. This dose pair allowed us to test both a diet‐achievable level and a supra‐dietary level.

Using standard dose conversion between animals and humans (HED (mg/kg) = animal dose (mg/kg) × (animal Km/human Km), with Km values of 3 for mice and 37 for humans)(Muñoz‐Esparza et al. 2019; Nair and Jacob 2016), the daily dose of 30 mg/kg in mice corresponds to a human equivalent dose (HED) of approximately 2.43 mg/kg/day, or about 146 mg daily for a 60 kg adult. Notably, the low dose of 5 mg/kg in mice gives an HED of approximately 0.4 mg/kg/day (24 mg/day for a 60 kg adult), which also showed a trend of alleviating intestinal injury and significantly prolonged the median survival time of irradiated mice. This low HED can be achieved through a diversified diet rich in SPD‐containing foods, suggesting that daily dietary SPD intake may help prevent radiation‐induced intestinal injury without the need for supplementation. While the high‐dose HED (146 mg/day) would be difficult to obtain from diet alone, even with a combination of SPD‐rich foods, it could be achieved through moderate dietary supplementation on top of a regular diet. Such supplementation would theoretically provide superior radioprotective effects compared to diet‐achievable intake alone, as suggested by the dose‐dependent protection observed in our animal study. Therefore, achieving a beneficial intake through a consciously planned diet, possibly aided by functional foods or modest supplementation, represents a highly practical and safe strategy for RIII management, with the supplemented higher dose offering potentially enhanced efficacy.

For SPD to be considered as a radioprotective agent for patients undergoing abdominal radiotherapy, its potential impact on tumor response would ideally be evaluated. However, the present study focused solely on normal intestinal protection and did not assess tumor‐bearing models—a clear limitation. Nevertheless, as SPD belongs to the polyamine family, its intriguing and somewhat perplexing dual role in cancer warrants brief discussion. Sustained activation of endogenous polyamine synthesis or long‐term high‐dose SPD exposure has been reported to promote tumor cell proliferation (Arruabarrena‐Aristorena et al. 2018; Prasher et al. 2023; Suzuki et al. 2025), whereas, in a striking contrast, dietary SPD may exert anti‐cancer effects via gut microbiota modulation (Prasher et al. 2023). Importantly, our regimen (short‐term, oral, relatively low dose) differs fundamentally from those pro‐tumor conditions. Moreover, our 16S rRNA data showed enrichment of butyrate‐producing bacteria (e.g., *Lachnospiraceae_NK4A136_group*) and suppression of pro‐inflammatory pathogens, changes generally associated with reduced cancer risk. Thus, under our experimental conditions, SPD does not raise an overt safety concern. Nonetheless, direct evaluation in tumor‐bearing models remains necessary before clinical translation, as noted in the Limitations section.

## 5 Limitations and Future Directions

This study has several limitations. First, the causal role of gut microbiota in SPD's effects was not established, as we did not measure SCFAs or perform FMT. Second, the study was conducted only in non‐tumor‐bearing models; whether SPD affects tumor radiosensitivity remains unknown. Third, our findings are preclinical and require validation in clinical trials. Future studies should address these gaps through metabolomics, FMT, and clinical translation.

## 6 Conclusion

Our findings establish that SPD confers multi‐targeted protection against radiation‐induced intestinal injury by coordinately regulating the “microbiota‐barrier‐inflammation” axis. SPD activates the Nrf2 pathway to alleviate oxidative stress and DNA damage, enhances tight junction protein expression to restore intestinal barrier integrity, and selectively remodels the gut microbiota by enriching beneficial bacteria while suppressing pathogens. Through these synergistic mechanisms, SPD alleviates inflammation, promotes epithelial repair, and ultimately enhances survival in irradiated animals. Our work provides experimental evidence for developing SPD as a novel radioprotective agent targeting microbe‐host interactions.

## Author Contributions

**Song Li:** writing – original draft, investigation, data curation, formal analysis, visualization. **Minglin Jiang:** investigation. **Yanjiang Liu:** investigation. **Kejian Pan:** funding acquisition, supervision. **Xiaolin Ren:** writing – review and editing, writing – original draft, supervision, visualization. **Hongtao Luo:** investigation. **Yu Zha:** investigation. **Chang Liu:** investigation. **Tao Zhang:** funding acquisition, supervision, methodology, conceptualization.

## Funding

This work was supported by the Scientific Research Fund of Chengdu Medical College (Grant No. CYZZD25‐19) and the Organized Research Fund of Chengdu Medical College (Grant No. CYYZZ25‐01).

## Ethics Statement

All animal experiments were approved by the Animal Ethics Committee of Chengdu Medical College (Approval No. [2025] 64; approval date: July 27, 2025). This approval specifically covered the experimental procedures described in this study, including total abdominal irradiation, SPD administration, and subsequent tissue, blood, and fecal sample collection. Animal care and experimental protocols were conducted in accordance with the national regulations and institutional guidelines for animal care and use in China.

## Conflicts of Interest

The authors declare no conflicts of interest.

## Supporting information


**Figure S1:** Stacked bar charts of microbial community composition at different taxonomic levels.
**Figure S2:** Heatmap analysis of the 30 most significantly altered OTUs.

## Data Availability

The supplementary data for this article, including the sequencing data, are available from the Science Data Bank at the following link: https://doi.org/10.57760/sciencedb.30706.
