# Supplementary material for: Dietary Spermidine Mitigates Radiation‐Induced Intestinal Injury by Reshaping the Microbiota‐Barrier‐Inflammation Axis
Source: Food Sci Nutr. 2026 Jun 18;14(6):e71924. doi: 10.1002/fsn3.71924 (PMC13279875; doi:10.1002/fsn3.71924)
Supplement: Supplementary file 1 — Figure S1: Stacked bar charts of microbial community composition at different taxonomic levels. Figure S2: Heatmap analysis of the 30 most significantly altered OTUs. [file FSN3-14-e71924-s001.docx]

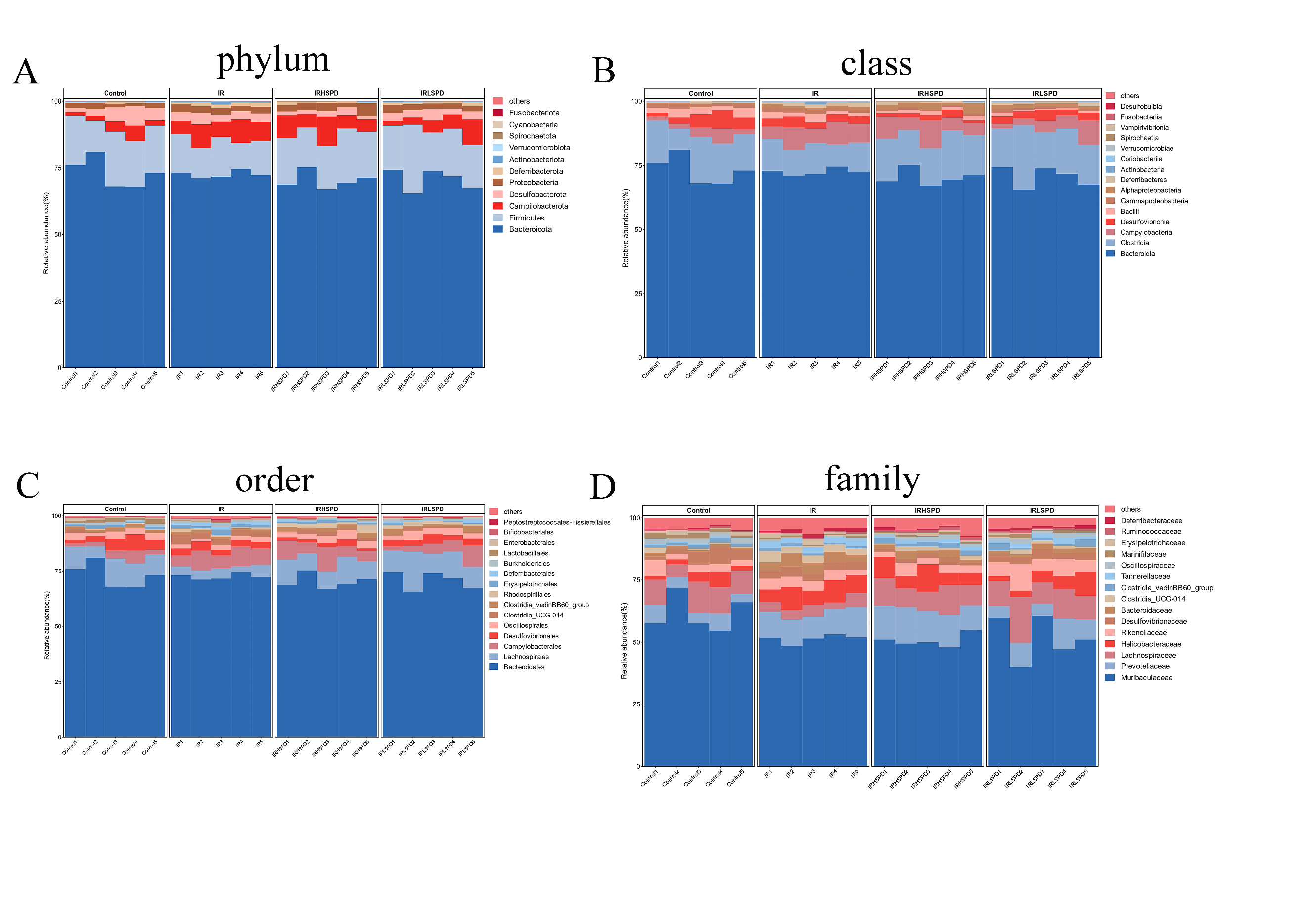


**Supplementary Figure 1. Microbial community composition at multiple taxonomic levels.**

Stacked bar charts illustrate the community structure at the **(A)** class, **(B)** order, **(C)** family and **(D)** genus levels, demonstrating that SPD treatment reverses the radiation-induced dysbiosis.


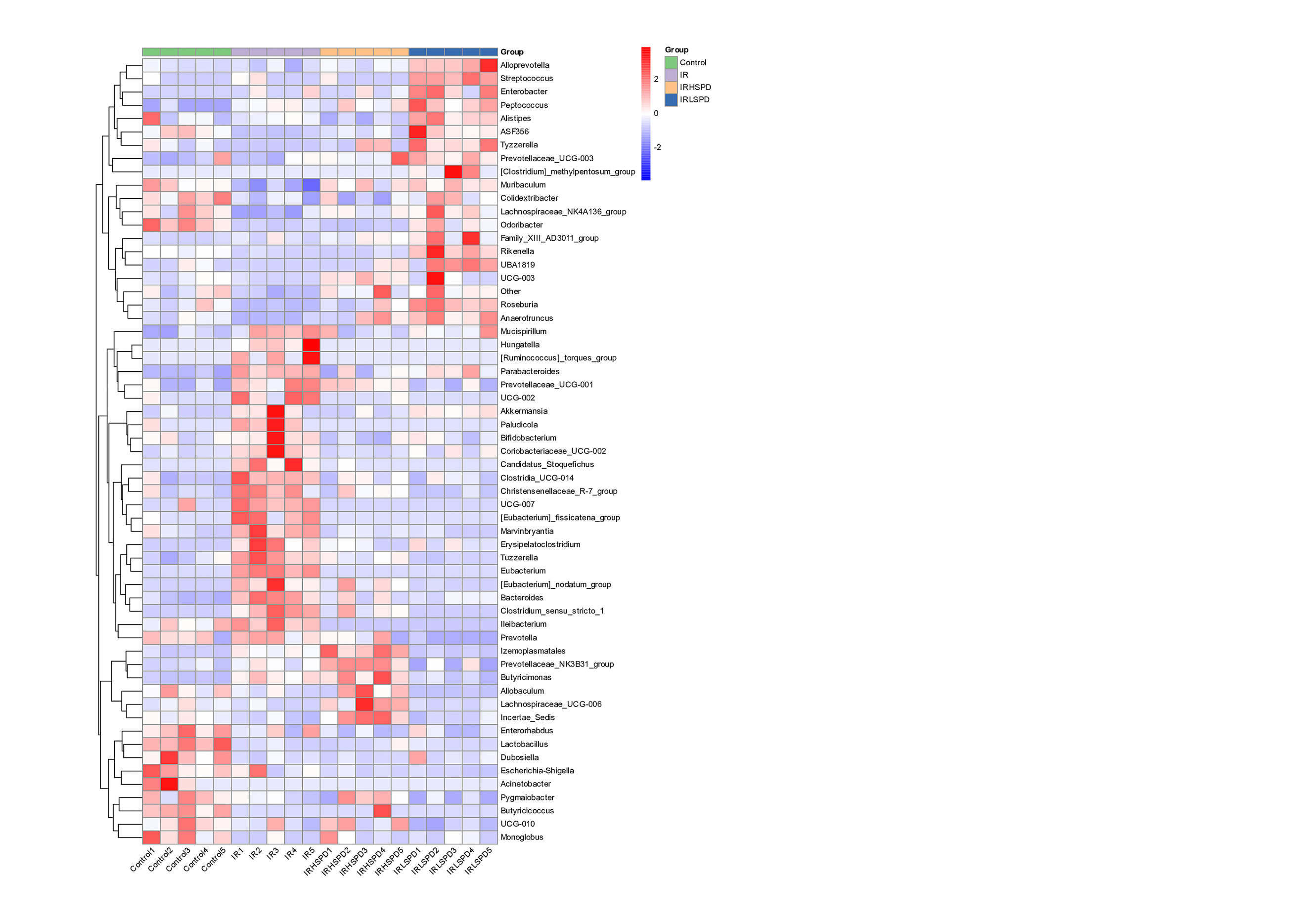


**Supplementary Figure 2. Heatmap of altered OTUs at the genus level.**

Heatmap of the 30 most significantly altered OTUs at the genus level, illustrating the specific dual mechanism of SPD.
